# Genome Sequence of *Bradyrhizobium mercantei* Strain SEMIA 6399^T^, Isolated from Nodules of *Deguelia costata* in Brazil

**DOI:** 10.1128/genomeA.00943-17

**Published:** 2017-09-07

**Authors:** Renan Augusto Ribeiro, Luisa Caroline Ferraz Helene, Jakeline Renata Marçon Delamuta, Mariangela Hungria

**Affiliations:** a Conselho Nacional de Desenvolvimento Científico e Tecnológico (CNPq), Brasília, Brazil; b Embrapa Soja, Londrina, Brazil; c Universidade Estadual de Londrina (UEL), Department of Biochemistry and Biotechnology, Londrina, Brazil; d Coordenação de Aperfeiçoamento de Pessoal de Nível Superior (CAPES), Brasília, Brazil

## Abstract

SEMIA 6399^T^ is the type strain of *Bradyrhizobium mercantei*, a nitrogen-fixing symbiont of *Deguelia costata*. Its draft genome contains 8,842,857 bp with 8,246 predicted coding sequences (CDS), several related to amino acids and derivatives and to stress tolerance, with an emphasis on oxidative stress, in addition to symbiotic genes.

## GENOME ANNOUNCEMENT

Bacteria known as rhizobia are capable of establishing symbioses with a broad range of legumes, resulting in the nitrogen fixation process, with high impact on agriculture productivity and environmental sustainability. In the tropics, *Bradyrhizobium* bacteria are the predominant symbionts of legumes (1–3). The genus occupies a variety of ecosystems and is enriched in living styles (1–4), and in the past few years, our group has reported large genetic diversity among Brazilian *Bradyrhizobium* strains (2, 3), resulting in the description of new species within this genus (5–7).

Here, we report the draft genome of strain SEMIA 6399^T^ (=CNPSo 1165^T^ = BR 6010^T^ = U675^T^ =LMG 30031^T^) of the recently described new species *Bradyrhizobium mercantei* (8). The strain is a symbiont of *Deguelia costata* (syn. *Lonchocarpus costatus*), an important legume native to eastern Brazil; SEMIA 6399^T^ has been used in commercial inoculants for this legume in Brazil since 1994 (8). The species is named after Fábio Martins Mercante (1963 to 2016), an extraordinary Brazilian microbiologist from Embrapa who dedicated his career to studies on biological nitrogen fixation (8).

To access the bacterial genome sequence, total DNA was extracted using the DNeasy blood and tissue kit (Qiagen) and processed on the MiSeq platform (Illumina) at Embrapa Soja, Londrina, Brazil. Shotgun sequencing allowed a genome coverage of approximately 29-fold. The FASTQ files were *de novo* assembled by the A5-MiSeq pipeline (9). The genome was estimated at 8,842,857 bp, assembled in 72 contigs, with a G+C content of 63.99 mol%. Average nucleotide identity (ANI) with the closest species, *B. tropiciagri* CNPSo 1112^T^, was 91.5%.

Sequences were submitted to RAST (10), and annotation identified 8,243 coding sequences (CDS). The analysis at the SEED system (11) allowed the classification of 41% of the CDS in 502 subsystems. Nitrogen fixation genes (*nif*) showed similarity with *Bradyrhizobium diazoefficiens* USDA 110^T^, and strain SEMIA 6399^T^ also possesses genes coding for the hydrogenase that may improve the efficiency of the nitrogen fixation process (12). Interestingly, the major category of putative genes was of the metabolism of amino acids and derivatives (15.3%), followed by the carbohydrates (14.5%). The genome is enriched with genes of resistance to antibiotics and toxic compounds (117 CDS) and genes of type I, II, III, and IV secretion systems, and it carries 214 CDS of stress response, the majority (55.1%) of which are related to the oxidative stress metabolism.

### Accession number(s).

This whole-genome shotgun project has been deposited at DDBJ/EMBL/GenBank under accession number MKFI00000000. The version described in this paper is MKFI01000000.
